# T-cell Receptor Excision Circles in Newborns with Heart Defects

**DOI:** 10.1007/s00246-020-02317-y

**Published:** 2020-03-13

**Authors:** Kiran A. Gul, Janne Strand, Rolf D. Pettersen, Henrik Brun, Tore G. Abrahamsen

**Affiliations:** 1 Department of Paediatric Research, Division of Paediatric and Adolescent Medicine, Oslo University Hospital Rikshospitalet, Oslo, Norway; 2 Norwegian National Unit for Newborn Screening, Division of Paediatric and Adolescent Medicine, Oslo University Hospital Rikshospitalet, Oslo, Norway; 3 Department of Paediatric Cardiology, Division of Paediatric and Adolescent Medicine, Oslo University Hospital Rikshospitalet, Oslo, Norway; 4 Centre for Rare Disorders, Division of Paediatric and Adolescent Medicine, Oslo University Hospital, Oslo, Norway; 5 Faculty of Medicine, University of Oslo, Oslo, Norway

**Keywords:** T-cell receptor excision circles, T-cell lymphopenia, Conotruncus, Conotruncal heart defects, Neural crest, 22q11.2 deletion syndrome

## Abstract

In the fetus, the cardiac neural crest gives rise to both the thymus and the conotruncus of the heart. In newborn screening for severe T-cell lymphopenia neonates with congenital heart defects may be detected. In this study, we investigated the occurrence of T-cell lymphopenia in neonates with or without 22q11.2 deletion syndrome (del) suffering from heart defects. This retrospective cohort study included 125 patients with heart defects. T-cell receptor excision circles (TRECs), a measure for T-cell lymphopenia, were quantified by RT-PCR using stored newborn screening blood spots. Three patient groups were compared: non-conotruncal defects (*n* = 57), conotruncal defects (*n* = 42), and 22q11.2 del with conotruncal defects (*n* = 26). Significantly lower TREC values were detected in patients with 22q11.2 del and conotruncal heart defects compared to those with non-syndromic conotruncal (*p* < 0.001) and non-conotruncal (*p* < 0.001) defects. In contrast, no significant difference was found between patients with non-syndromic conotruncal and non-conotruncal heart defects (*p* = 0.152). Low TREC levels were obtained in neonates treated with heart surgery/intervention within 2 weeks after birth and in those with a fatal outcome (*p* = 0.02) independent of patient group. A correlation was found between low TREC numbers and oxygen saturation, SpO_2_ below 95% (*p* = 0.017). The SpO_2_ was significantly lower in the non-syndromic conotruncal group compared to non-conotruncal (*p* < 0.001) and 22q11.2 del group (*p* = 0.015). No correlation was found between low neonatal TRECs and infections needing hospitalization later in life (*p* = 0.135). Patients with 22q11.2 del and conotruncal defects have significantly lower TREC levels compared to patients with heart defects without this syndrome.

## Introduction

T-cell lymphopenia in neonates with congenital heart defects occurring as part of a syndrome or as a non-syndromic condition, has been described in a number of studies [1, 2]. Newborn screening (NBS) for severe combined immunodeficiency (SCID) is now performed routinely in the USA and other countries including Norway. In this screening, both SCID and non-SCID conditions with T-cell lymphopenia are detected when determining T-cell receptor excision circles (TRECs). After screening 3 million newborns in ten American States, Kwan et al. [1] found an incidence of non-SCID T-cell lymphopenia of 1 in 14,000. Heart defects were found in 7% of these patients [1]. Congenital syndromes associated with T-cell lymphopenia, as the 22q11.2 deletion syndrome (del) and trisomy 21 (Down syndrome) were reported in 33% of these infants.

The incidence of congenital heart defects is approximately 1 in 100 [3]. Conotruncal heart defects, also known as outflow tract defects, represent 10–15% [3, 4]. They include severe conditions such as transposition of the great arteries, tetralogy of Fallot (TOF), double outlet right ventricle, interrupted aortic arch (IAA), pulmonary atresia (PA), truncus arteriosus (TA), and Conoventricular septal defects [5]. The conotruncal defects are often found in chromosomal syndromes [6] with 22q11.2 del occurring in 18% [7]. In a previous study, we tested 46 patients with 22q11.2 del and found that those with the lowest neonatal TREC numbers had the most severe heart defects [8]. In the present study, we wanted to compare the newborn TREC values in patients with non-conotruncal, non-syndromic conotruncal, and 22q11.2 del with conotruncal heart defects in order to study the effect of this chromosome microdeletion on the T-cell lymphopenia.

## Materials and Methods

### Patients and Samples

The patients invited to the study were identified through a registry of heart defects at the Oslo University Hospital. Our hospital has a national service for children with congenital heart defects which includes preoperative assessment and definitive treatment. Invitation letters were sent to 230 parents of children with heart defects, and 140 (61%) consented to participate. The rest did not respond to the invitation. One patient with Noonan’s syndrome and one with Turner’s syndrome were excluded from the study. In two patients, no screening card was available. In the non-syndromic conotruncal heart defect group, only patients with a negative genetic test for 22q11.2 del were included. We finally studied 125 patients born after 2005. They were diagnosed with non-syndromic conotruncal (*n* = 42), non-conotruncal (*n* = 57), or 22q11.2 del with conotruncal heart defects (*n* = 26). The 22q11.2 deletion was diagnosed by fluorescent in situ hybridization (FISH, *n* = 8), multiplex ligation-dependent probe amplification (MLPA, *n* = 16), or comparative genomic hybridization array (CGH array, *n* = 2).

Filter cards stored at a temperature between − 20 °C and − 25 °C were retrieved from the National newborn screening diagnostic biobank (Oslo University Hospital, Norway).

Patient medical records were obtained from the hospitals, and a review was conducted by the first author. Data were collected, including the type of cardiac defects, treatment procedure (surgery/ intervention), gestational age, delivery mode, the identification of a syndrome diagnosis, and information about hospitalization due to infection later in life. SpO_2_ values before operation/intervention, measured by pulse oximetry, were also retrieved.

### TREC Test

We used TREC levels as marker for T-cell lymphopenia [1, 9]. TRECs are circular DNA fragments generated during the sequential rearrangement of variable V, D, and J segments of T-cell receptor (TCR) genes [10]. These DNA circles do not replicate and will, therefore, be diluted after each cell division of T cells when they have emigrated from the thymus [11].

### Extraction of DNA

DNA extraction was performed in a 96-well format. One 3.2 mm dried blood spot of each patient’s screening card was punched into a 96-well PCR plate (VWR) using a Panthera puncher (PerkinElmer). 150 µL Generation DNA elution solution (Qiagen) was added to each well, and the samples were incubated at 60 °C for 10 min on a microplate shaker (Eppendorf) at 1000 rpm. The supernatant was discarded, and the DNA was extracted in 100 µL Generation DNA elution solution by incubation at 100 °C for 30 min in a PCR instrument.

### TREC qRT-PCR

TREC and β-actin qPCR reactions were run in final volumes of 20 μL containing 10 µL PerfeCTa qPCR ToughMix (Quantabio), 500 nmol/L forward and reverse primers, 150 nmol/L probe, 0.4 mg/mL BSA and 8 µL DNA (4 µL for the β-actin assay). The primer sequences are provided in Table 1. The reactions were carried out on a ViiA™7 or QuantStudio7 Real-Time PCR system (Applied Biosystems) with the following program: 50 °C for 2 min, 95 °C for 10 min followed by 45 cycles of 30 s at 95 °C and 60 s at 60 °C.

**Table 1:** Primers and probes used in the RT-PCR reactions

TREC forward	5′-CAC ATC CCT TTC AAC CAT GCT-3′
TREC reverse	5’GCC AGC TGC AGG GTT TAG G-3′
TREC probe	5′-FAM-ACA CCT CTG GTT TTT GTA AAG GTG CCC ACT-3′ TAMRA
β-actin forward	5′-ATT TCC CTC TCA GGC ATG GA-3′
β-actin reverse	
	5′-CGT CAC ACT TCA TGA TGG AGT TG-3′
β-actin probe	5′- FAM-GTG GCA TCC ACG AAA CTA-3′-TAMRA

Copy numbers were calculated based on standard curves generated from serially diluted plasmids kindly provided by Douek [12]. All the qPCR assessments fulfilled the quality requirements of similar slopes and with *R*^2^ values > 0.99. β-actin was used as a housekeeping gene to assure an adequate DNA extraction for PCR. Quality control was performed using TREC NBS QC provided by Center of Disease Control. The TREC value per μL was calculated assuming that a 3.2 mm punch contains ∼3 μL of blood.

### Statistical Analysis

One-way ANOVA was performed with post hoc Tukey test to compare TREC values or SpO_2_ values below 95% in patients with non-syndromic conotruncal, non-conotruncal, and 22q11.2 del with conotruncal defects. The correlation between TREC groups (TREC quartiles) and heart surgery/intervention within 2 weeks of life or death was done by using crosstabs and Chi-square Linear by linear association. For other clinical comparisons, student T test was employed. A significance level of 5% was used. All figures were created using Graphpad prism.

## Results

### TREC Values in Different Groups of Heart Defect

**Fig. 1:**
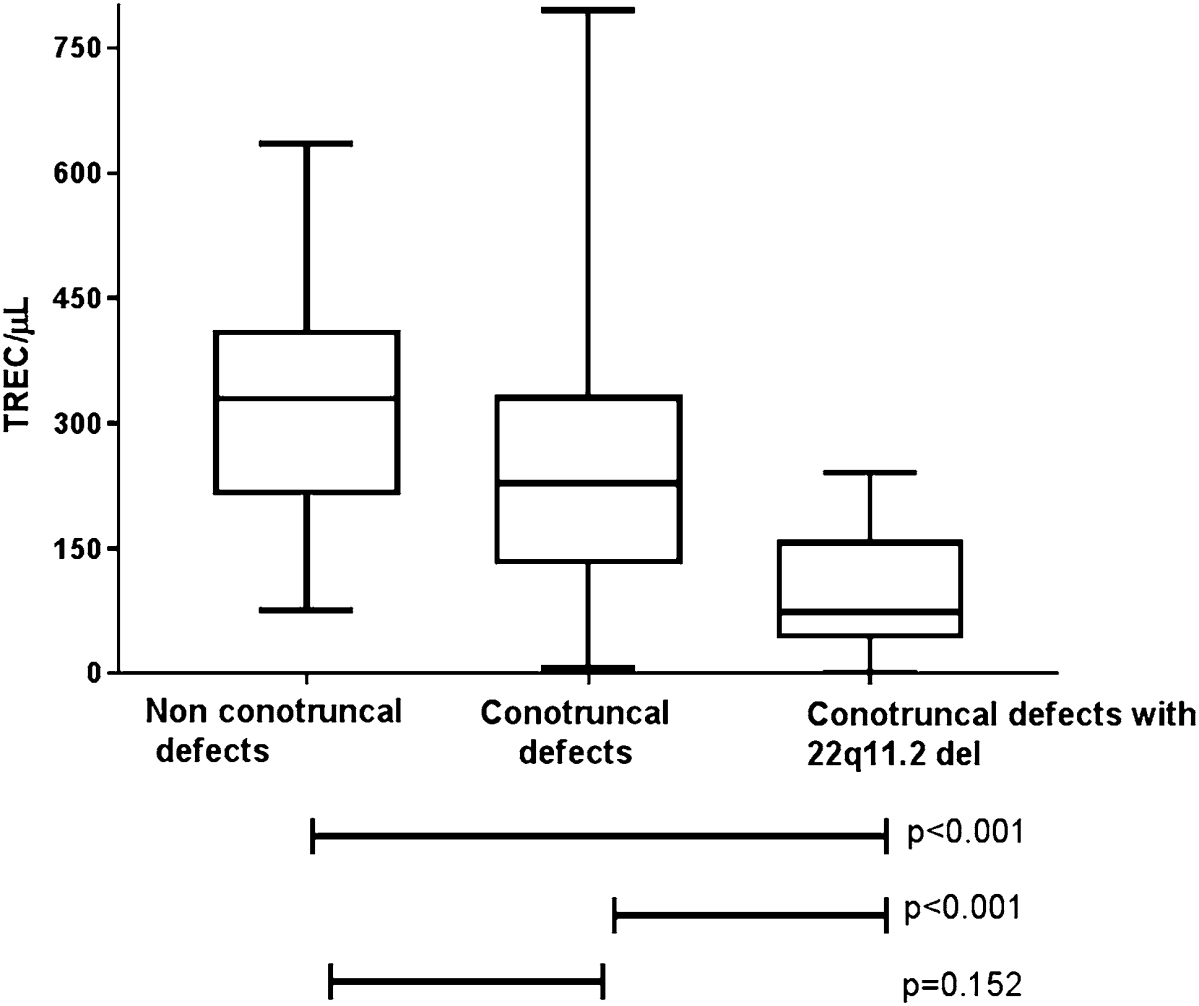
TREC values in patients with heart defects

The patients were divided in three groups, those with non-conotruncal (*n* = 57), conotruncal (*n* = 42), and 22q11.2 del with conotruncal heart defects (*n* = 26). The number of patients and the specified diagnoses are listed in Table 2. The mean number of TRECs was 323/μL in the non-conotruncal group, 270/μL in the conotruncal group, and 94/μL in the 22q11.2 del group (Fig. 1, Table 3). Significantly lower number of TREC (*p* < 0.001) was found in the 22q11.2 del group compared to both non-syndromic conotruncal and to non-conotruncal heart defect groups. In the non-syndromic patients, a slight trend of lower TREC values was observed in the conotruncal group compared to non-conotruncal although not significant (*p* = 0.152). Lower TRECs were found in the 22q11.2 del group compared to the non-syndromic conotruncal group when investigating specific diagnoses such as TOF (*p* < 0.001), PA (*p* = 0.017), and IAA (*p* = 0.066).

**Table 2:** TREC values in different heart defects

	Non-conotruncal	TREC/µL^a^	Conotruncal	TREC/µL	22q11.2 del with conotruncal	TREC/µL
Heart defects	Atrioventricular septal defect (*n*^b^ = 1)	252	Truncus arteriosus (*n* = 3)	261 (134–363)	Truncus arteriosus (*n* = 1)	74
	Atrial septal defect (*n* = 25)	330 (110–595)	Transposition of the great arteries (n = 19)	289 (75–795)	Simple septal defects (*n* = 4)	143 (84–241)
	Valvular pulmonary stenosis (*n* = 14)	308 (124–635)	Tetralogy of fallot (*n * = 5)	285 (238–380)	Tetralogy of fallot (*n* = 8)	71 (23–215)
	Aortic stenosis (*n* = 17)	331(75–627)	Pulmonary atresia with ventricular septal defect (*n* = 5)	230 (138–326)	Pulmonary atresia with ventricular septal defect (*n* = 4)	74 (84–161)
			Pulmonary atresia without ventricular septal defect (*n* = 3)	250 (189–317)	Pulmonary atresia without ventricular septal defect (*n* = 1)	53
			Interrupted aortic arch (*n* = 6)	275 (7–548)	Interrupted aortic arch (*n* = 6)	113 (0–190)
			Congenital corrected transposition of the great arteries (*n* = 1)	102	Absent pulmonary valve, overriding aorta, ventricular septal defect (*n* = 1)	84

^a^Mean and range in each diagnosis

^b^Number of patients

**Table 3:** Characteristics of the patient cohorts with heart defects

	Non-conotruncal	Conotruncal	22q11.2 del with conotruncal
Mean TREC	323 TREC/µL^a^	270 TREC/µL	94 TREC/µL
Operated within 14 days	10^b^	35	12
Died	0	3	4
Hospitalization due to infections	13/47^c^	17/31	14/25

^a^Mean values in each group

^b^Number of patients

^c^Number of patients with information available

### TREC Values and Heart Surgery/Intervention/Fatal Outcome

In line with our previous study [8], the risk of death or heart surgery/intervention during the first 2 weeks of life decreased with increasing TREC values (*p* = 0.02). A similar, but not so strong correlation was found if 22q11.2 del patients were excluded from the calculation (*p* = 0.041).

In 101 patients, we were able to collect neonatal SpO_2_ values preferably the first measurement after birth and obtained before heart surgery/intervention. A correlation was found between low TREC and SpO_2_ below 95% (*p *= 0.017). The SpO_2_ was significantly lower in the conotruncal non-syndromic group (*p* < 0.001) and 22q11.2 del group (*p* = 0.002) compared to non-conotruncal heart defects group. In the conotruncal non-syndromic group, the SpO_2_ was significantly lower (*p* = 0.015) than in 22q11.2 del.

### TREC Values and Prematurity, Birth Weight, and Delivery Mode

Twelve of the patients were premature (Table 4), born in week 31–36. They had non-conotruncal (*n* = 6), conotruncal (*n* = 1) or 22q11.2 del with conotruncal (*n* = 5) heart defect, respectively. Exclusion of these patients gave similar TREC results (data not shown). Also, no association was found between TRECs and birth weight (below or above 2500 g, data not shown) or delivery mode (*n* = 110, vaginal vs cesarean).

**Table 4:** TREC values in premature infants, heart defect type, and diagnosis

Heart defects	Premature infants	Gestational age	TREC values/ µL	Diagnosis
Non-conotruncal	1	34	120	ASD
	2	34	110	ASD
	3	36	220	VPS
	4	35	234	ASD
	5	34	302	ASD
	6	31	595	ASD
Conotruncal	7	36	102	CCTGA
Conotruncal with 22q11.2 del	8	34	161	PA, VSD
	9	36	141	IAA
	10	35	36	IAA
	11	34	53	PA, VSD
	12	33	40	TOF

*ASD* atrial septum defect, *CCTGA* congenital corrected transposition of the great vessles, *IAA* interrupted aortic arch, *PA* pulmonal atresi, *TA* truncus arteriosus, *TOF* triade of Fallot, *VSD* ventricular septum defect, *VPS* valvular pulmonal stenosis

### TREC Values and Infections

No association (*n* = 103) was found between the newborn TREC value and serious infections occurring later in life. The number of patients hospitalized due to infections is presented in Table 3. These patients were up to 8 years old. No association was found.

## Discussion

### TREC Values in Different Heart Defects

We found significantly lower number of TRECs in 22q11.2 del patients with conotruncal heart defects compared to patients with conotruncal and non-conotruncal defects without this syndrome. Non-syndromic patients with conotruncal defects had a tendency, although not significant, of lower TREC values than those with non-conotruncal defects. In line with our results Sullivan et al. [13] found lower absolute lymphocyte counts and CD3, CD4 and CD8 T-cell counts in neonates (median age 17 days) with 22q11.2 del and cardiac anomalies when compared to controls with cardiac defects.

We also compared the TREC values in neonates with and without the syndrome, suffering from different groups of conotruncal heart defects (TOF, PA and IAA). We found significantly lower TREC values in TOF and PA patients with 22q11.2 del. Sullivan et al. [14] studied 43 children (above 6 months of age) with 22q11.2 del and 5 distinct cardiac anomalies and found no correlation between T-cell lymphopenia and the specific cardiac anomaly. In another study [15] they investigated a larger group of patients (*n* = 353) with 22q11.2 del. They found no correlation between T-cell numbers and the severity of cardiac anomaly (divided in three groups, according to need for operation) or the complexity of the surgical procedure.

In this respect the common embryology of the heart and the thymus is interesting. The thymus develops from the 3rd pharyngeal pouch endoderm and cardiac neural crest cells [16, 17]. The latter cells also give rise to the outflow tract of the heart with aortic arches and the aorta and pulmonary arteries [18]. Kirby et al. [19, 20] demonstrated that ablation of the cardiac neural crest in chick embryos leads to hypoplasia/aplasia of the thymus, defective development of the cardiac outflow tract resulting in conotruncal defects. Also in mice disruption of the cardiac neural crest migration causes many of the 22q11.2 del features such as thymus hypoplasia/aplasia and conotruncal heart defects [21]. Patients with 22q11.2 del usually have a haploinsufficiency of the TBX1 gene [22]. This gene encodes a transcription factor which is important for the migration of the neural crest cells into the 3rd and 4th pharyngeal arches and pouches.

### TREC Values and Heart Surgery/Intervention/Fatal Outcome

Dar et al. [23] compared 22q11.2 del patients with and without heart surgery and found no correlation between low TREC levels and need for heart surgery. However, in our study we found an increased risk of heart surgery/intervention or death within 2 weeks after birth in patients with lowest TREC values. Neonates requiring early heart surgery/intervention or having a fatal outcome may have physical and emotional stress that activates the hypothalamus–pituitary–adrenal (HPA) axis. This may lead to an increased production of cortisol and cause thymus involution and reduction in thymopoiesis [24, 25].

In animal models, cyanotic heart disease causing hypoxia has been found to increase endogenous glucocorticoid production [26–28]. This may in part be the reason for the lower TRECs in the conotruncal heart defect group as compared to the non-conotruncal group. This is supported by our finding of low TRECs when SpO_2_ is below 95%. However, the SpO_2_ was significantly lower in non-syndromic conotruncal heart defects compared to patients with 22q11.2 del. This suggests that stress alone cannot explain the low TRECs in 22q11.2 del group, and a study of 29 infants with chronic hypoxia due to cyanotic congenital heart disease, did not show any effect on the HPA axis [29]. However, in the T-cell lymphopenia (TCL) screening, higher rates of abnormal screen are seen in the premature and critical ill infants [30]. Gerstel-Thompson [31] demonstrated that higher proportion of infants in neonatal intensive care units (NICUs) had lower TREC than non-NICU infants. Kwan et al. [30] described occurrence of non-SCID TCL in patients with congenital defects. This TCL improved when the defects were repaired. Suppression of the lymphopoiesis caused by stress may be one of the main reasons for TCL in this group [30]. A better marker for hypoxia could be serum lactic acid. Unfortunately we did not have such values available.

### TREC Values and Prematurity, Birth Weight, and Delivery Mode

Previous studies have shown a correlation between prematurity and low TREC levels [32, 33]. TREC usually normalize with increasing gestational age. In our study, excluding the premature infants did not affect our findings. Furthermore, low birth weight or mode of delivery did not influence the TREC values, in contrast to what was reported in healthy children by Schlinzig et al. [34].

### TREC Values and Infection

In 103 patients, we had information about later hospitalizations. As in our previous report [8], we did not find any association between low neonatal TREC levels for infections when they grew older. However, our study lacks information from general practitioners and parents about days off from day care institution/school. Therefore, we cannot totally exclude an association between infections in general and low TREC levels.

## Conclusion

In the present study we have demonstrated that neonates with 22q11.2 del and conotruncal heart defects have significantly lower TREC levels than non-syndromic patients with conotruncal or non-conotruncal defects. This result indicates that T-cell lymphopenia (e.g., low TREC values) observed in the conotruncal heart defects is mainly due to the chromosome 22q11.2 deletion. However, the stress due to the conotruncal heart defects in either group may also affect thymus function and result in low TREC values.

## Abbreviations

ASD: Atrial septal defect
CGH array: Comparative genomic hybridization array
Del: Deletion syndrome
FISH: Fluorescent in situ hybridization
IAA: Interrupted aortic arch
NBS: Newborn screening
NICUs: Neonatal intensive care units
PA: Pulmonary atresia
SCID: Severe combined immunodeficiency
TA: Truncus arteriosus
TCR: T-cell receptor
TCL: T-cell lymphopenia
TOF: Tetralogy of Fallot
TREC: T-cell receptor excision circle
VSD: Ventricular septal defect
SpO_2_: Oxygen saturation
